# Performance comparison in workflow efficiency between a remotely installed 3D workstation and an on-premises image processing workstation for dental cone-beam CT image reconstruction

**DOI:** 10.1007/s11282-025-00806-5

**Published:** 2025-01-24

**Authors:** Ryoichi Tanaka, Hiroki Mouri, Noriaki Takahashi, Mitsuru Izumisawa, Masayuki Hoshino, Riku Sakamoto, Takaki Kanamori, Ami Shimamura, Ryota Sakai, Emi Kanno, Motoi Sawano

**Affiliations:** Division of Dental Radiology, Department of Reconstructive Oral and Maxillofacial Surgery, School of Dentistry, Iwate Medical University, 19-1 Uchimaru, Morioka, Iwate 020-8508 Japan

**Keywords:** CBCT, 3D image reconstruction, Multi-planar reformation, Remote display protocol, Processing time comparison

## Abstract

**Objectives:**

This study aims to compare the image processing times of dental cone beam CT (CBCT) images using a remote medical image processing workstation (RW) versus on-premises image processing (OP) and assess its impact on workflow efficiency.

**Methods:**

Data from 100 CBCT cases were randomly selected and processed using the OP3D VISION 17-19DX (EH Japan Co., Ltd.). In the OP environment, OnDemand 3D Dental (Cybermed Inc.) was used on a local terminal, while the RW setup involved a remote workstation—ZIO STATION (Ziosoft Inc.) connected via a 2 Gbps network. Seven experienced dentists processed the same data in both environments, and various processing times, including data transfer, re-slicing, 3D reconstruction, and PACS transfer, were compared.

**Results:**

The RW environment showed significantly shorter data transfer and re-slicing times than the OP environment. However, 3D image reconstruction times were similar between the two setups. Overall, processing time was significantly reduced in the RW environment. Variability in processing times among operators was observed, with most achieving reductions in the RW environment.

**Conclusions:**

Remote processing of dental CBCT images using a dedicated image processing device offers equivalent or improved performance compared to on-premises processing. This approach can enhance workflow efficiency by reducing processing times and freeing up local resources, although further research is needed to optimize remote display protocols and multi-client access.

## Introduction

Dental cone beam CT (CBCT) provides cross-sectional images of the jawbone and dental structures. It also allows for a detailed understanding of lesions and 3D anatomical structures through 3D image reconstruction using volume rendering. In addition, it offers cross-sectional images along the dentition and tooth axis using multi-planar and curved reconstruction. The reconstruction process uses many images with larger matrices than those of standard CT or MRI, resulting in a substantial image capacity [1]. As a result, the time required for image processing depends on the performance of the image processing device and network.

Image processing often depends on the software and processors that come with the imaging device. However, image processing performance can vary based on the configuration of the imaging device and image processor. In addition, the processor acts as a gateway for data transfer, which adds to the processing time and can be burdensome. Medical image processors excel in processing speed and handle a wide range of image processing tasks [2], potentially leading to performance improvements. They also do not take up space on the terminal connected to the imaging device, which can help improve workflow.

### Purpose

This study aims to compare the processing time of dental CBCT images when using a remotely located medical image processing workstation with a remote display protocol (RW) with the processing time for on-premises image processing workstation (OP) and examine whether it contributes to workflow improvement.

## Materials and methods

**Fig. 1:**
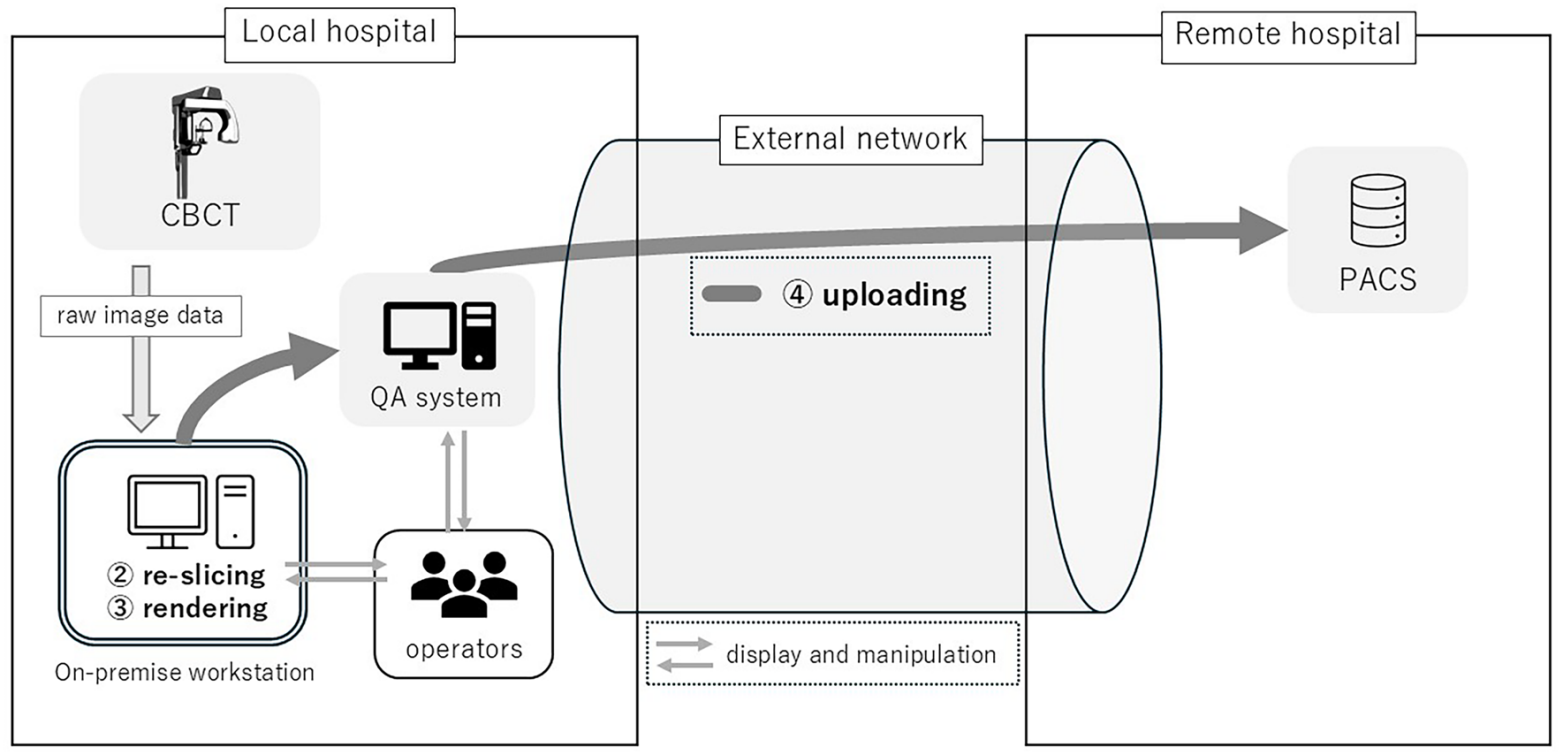
Data flow in the OP system and measurement points. The system overview illustrates the relationship between the local and remote systems. An external network exchanges actual image data between the local and remote hospitals. In addition, the screen data of the remote workstation and the operator's manipulation data are transmitted through the same network. The measurement points include ② re-slicing, ③ rendering, and ④ uploading to the PACS after the quality assessment of the images, as ① transferring between workstations did not occur in the OP system. On the local system, raw image data are automatically transferred to an on-premise workstation from the CBCT, eliminating the transfer time for step ①. *CBCT* cone beam computed tomography; *QA* quality assessment; *PACS* picture archiving and communication system

**Fig. 2:**
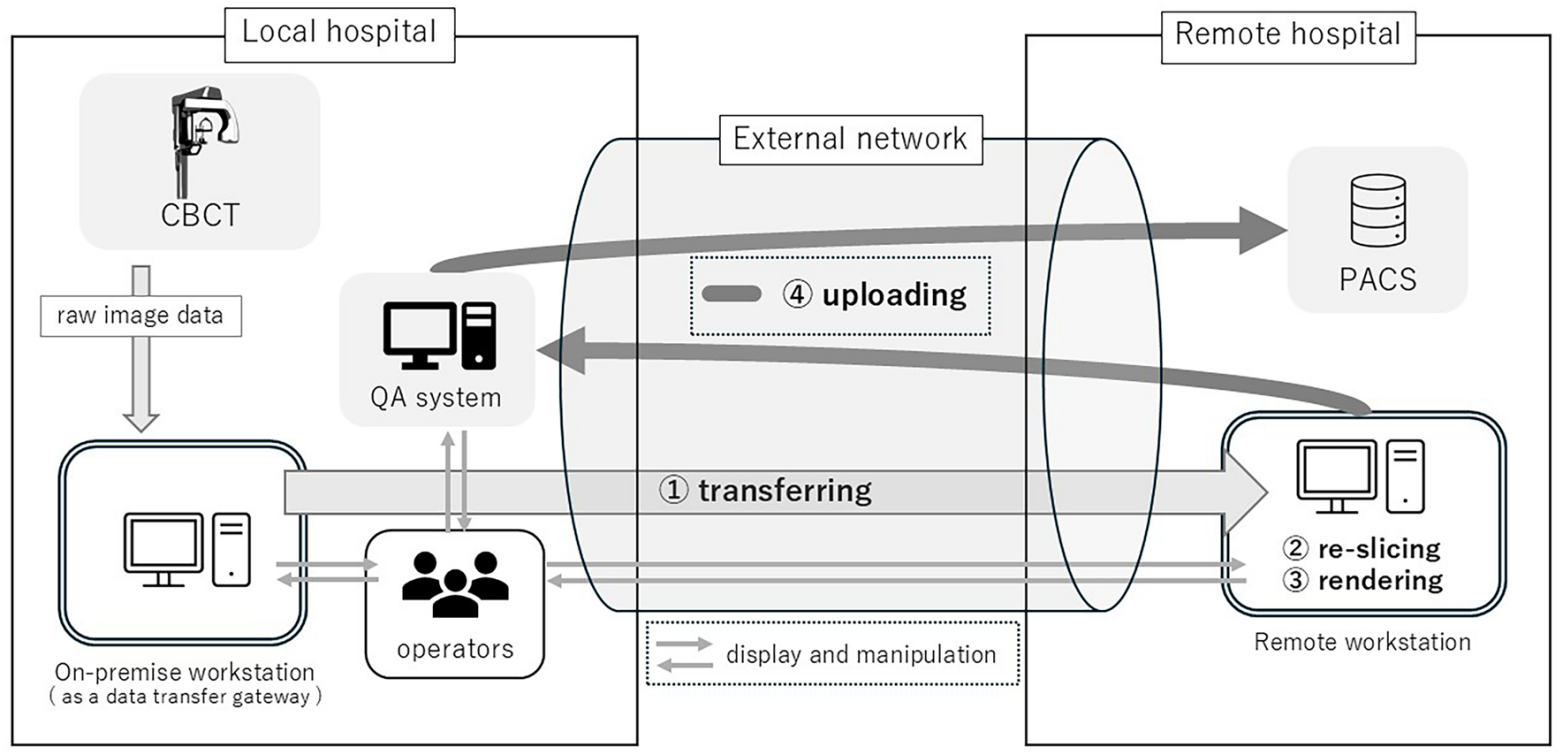
Data flow in the RW system and measurement points. The system overview illustrates the relationship between the local and remote systems. The measurement points include ① transferring, ② re-slicing, ③ rendering, and ④ uploading to the PACS after the quality assessment of the images. Due to manufacturer specifications, the QA process involves image data being temporarily transferred to the local system in the background. *CBCT* cone beam computed tomography; *QA* quality assessment; *PACS* picture archiving and communication system

The data for processing were randomly selected from 100 cases of CBCT performed between April and May 2022. The imaging device used was the OP3D VISION 17-19DX (EH Japan Co., Ltd., Tokyo). For image processing, the OP environment utilized a terminal installed with OnDemand 3D Dental (Cybermed Inc., Seoul), which is supplied with the dental CBCT and connected via LAN within the same area as the imaging device. In contrast, the RW setup used an image processing workstation—ZIO STATION (Ziosoft Inc., Tokyo) located 11 km away in a remote location. This workstation was connected to a local image-reading PC using a remote display protocol over a duplicated commercial network line with a total capacity of 2 Gbps (1 Gbps × 2) (Figs. 1 and 2).

The image-processing tasks were distributed among seven dentists with varying levels of experience as operators. The years of experience for each operator (1–7) were as follows: 20 years, 20 years, 1 year, 2 years, 2 years, 7 years, and 2 years. Each operator processed the same data using both the OP and RW environments to compare the time required for a series of functions.

The fields of view (FOV) used for imaging were 16 × 6, 16 × 8, and 16 × 10, selected based on the physique of the subjects.

The processing sequence included ① transferring: transferring and loading the original data, ② re-slicing: re-slicing for correcting the orientation of the subject during CBCT imaging, ③ rendering: loading the re-sliced data, performing three-dimensional image renderings, such as multiplanar reformation (MPR), curved planar reformation (CPR), and volume rendering (VR), and ④ uploading: transferring the images to the picture archiving and communication system (PACS). During the series of processes, the data transfer time to the image processing workstation, re-slicing time, image rendering time, and transfer time to PACS were measured (Figs. 1 and 2).

### Statistical analysis

Statistical analysis was performed using R [3].

Since there are no equivalent prior studies, the sample size was calculated based on the minimum number required for statistical analysis, considering the results of a preliminary sample. Specifically, assuming a difference of over 20 s between the two groups, the variance was calculated from the measurements of 10 cases to determine the effect size. The significance level was set at 0.05 and the power at 0.8. Taking into account the effects of the operator, FOV, and multiple comparisons, the sample size was determined to be 100 cases, which is close to the result calculated using the pwr.anova.test function.

Differences in data processing volume by FOV or operator were separately evaluated using the Kruskal–Wallis rank sum test.

The Kolmogorov–Smirnov test assessed the normality of the data distribution. The nonparametric Wilcoxon signed-rank test was employed to compare two paired groups, with multiple comparisons corrected using the Hochberg method. The significance level was set at 0.05.

The influence of each measured item, the size of the FOV, and the operator were set as explanatory variables. Multiple regression analysis was then performed in each group for evaluation.

## Results

A significant difference in data volume was observed depending on the size of the FOV (*p* = 3.765e-12). However, it was confirmed that there was no difference in the data volume processed among the operators (*p* = 0.9142) (Table 1).

**Table 1:** Data size characteristics in each FOV and operator

Variables	Mean (MB)	SD	Minimum (MB)	Maximum (MB)
FOV				
FOV 16 × 6	381.7	60.3	152.0	516.7
FOV 16 × 8	545.3	63.0	370.2	641.8
FOV 16 × 10	346.5	85.1	319.3	578.7
Kruskal–Wallis chi-squared = 52.611, df = 2, *p* = 3.765e-12				
Operator				
Operator 1	411.2	101.3	330.2	611.7
Operator 2	383.3	26.4	348.1	406.3
Operator 3	414.1	73.9	321.4	537.8
Operator 4	430.3	104.2	157.5	620.8
Operator 5	428.3	118.1	323.8	639.8
Operator 6	434.3	121.2	152.0	641.8
Operator 7	458.6	88.3	345.6	536.4
Kruskal–Wallis chi-squared = 2.059, df = 6, *p* = 0.9142				

**Fig. 3:**
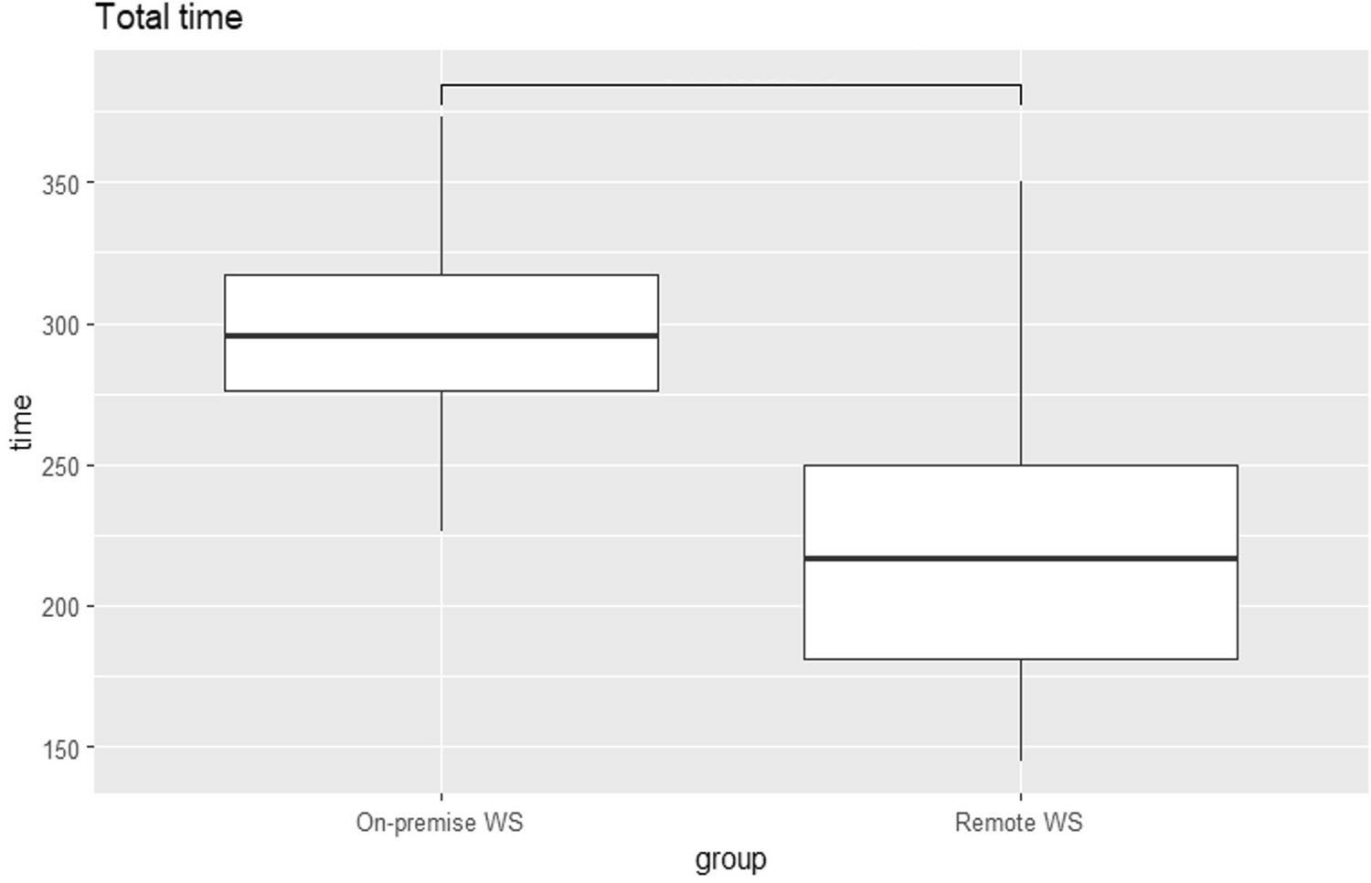
Difference in total processing time between OP and RW. In the RW environment, a significant processing time reduction was achieved

**Fig. 4:**
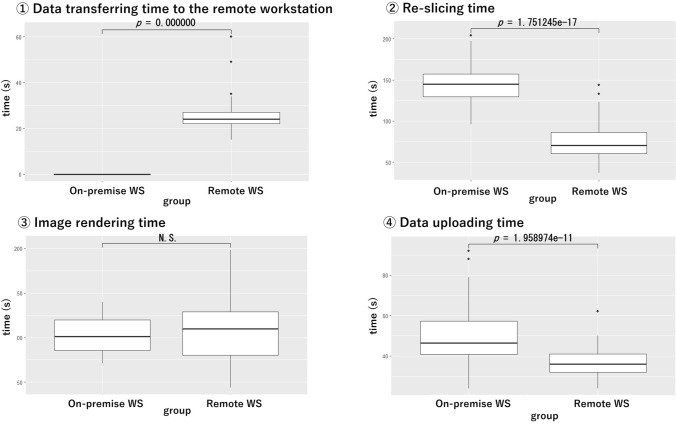
Comparison in time for each measurement points. The re-slicing (②) and data uploading (④) time processes were significantly reduced in the RW environment

The results are shown in Table 2, Figs. 3, and 4. Data transfer time has two components: the time for data transfer to the image processing workstation and the time for image transfer to the PACS. In the OP environment, the transfer time was 0, because the data were directly saved from the imaging device. In contrast, the RW environment required data transfer, resulting in an average transfer time of 25 s. The time needed to transfer reconstructed images to the PACS was significantly shorter in the RW environment (37 ± 7 s) compared to the OP environment (49 ± 13 s) (*p* = 1.958974e-11).

**Table 2:** Comparison of time required for OP and RW

	OP	RW	*p *value	Corrected *p *value
Total time (s)	296 ± 35	221 ± 49	8.118968e-15^***^	2.435690e-14^***^
Transferring	0 ± 0	25 ± 6	0.000000e + 00^***^	0.000000e + 00^***^
Re-slicing	145 ± 24	75 ± 22	4.378112e-18^***^	1.751245e-17^***^
Rendering	102 ± 20	110 ± 37	1.497074e-01	1.497074e-01
Uploading	49 ± 13	37 ± 7	9.794871e-12^***^	1.958974e-11^***^

Variables in OP and RW are expressed as mean ± SD

^***^indicates *p* < 0.05

For image processing, the re-slicing time was significantly shorter in the RW environment (75 ± 22 s) compared to the OP environment (145 ± 24 s) (*p* = 1.751245e-18). However, there was no significant difference between the two environments in the time required for 3D image reconstruction (*p* = 0.1497074). When comparing the total processing time, the RW environment was significantly shorter (221 ± 49 s) than the OP environment (296 ± 35 s) (*p* = 2.435690e-14).

Regarding the FOV during imaging, no significant correlation was found with processing time in the OP or RW environment (Table 3).

**Table 3:** Multiple regression analysis of FOV versus processing time in OP and RW

Variables	*β*	SE	*t*	*p*
Results in OP environment				
Independent variable 1 (FOV 16 × 6)	– 4.450	7.821	– 0.569	0.5707
Independent variable 2 (FOV 16 × 8)	30.401	8.681	3.502	0.0007
Intercept (FOV 16 × 10)	289.885	6.251	46.373	< 2e-16
Overall model: *R*^2^ = 0.1698, F(2, 97) = 11.13, *p* = 4.463e-05				
Results in RW environment				
Independent variable 1 (FOV 16 × 6)	8.978	13.469	0.667	0.507
Independent variable 2 (FOV 16 × 8)	– 6.438	12.134	– 0.531	0.597
Intercept (FOV 16 × 10)	221.808	9.699	22.870	< 2e-16
Overall model: *R*^2^ = -0.003098, F(2, 97) = 0.8471, *p* = 0.4318				

**Fig. 5:**
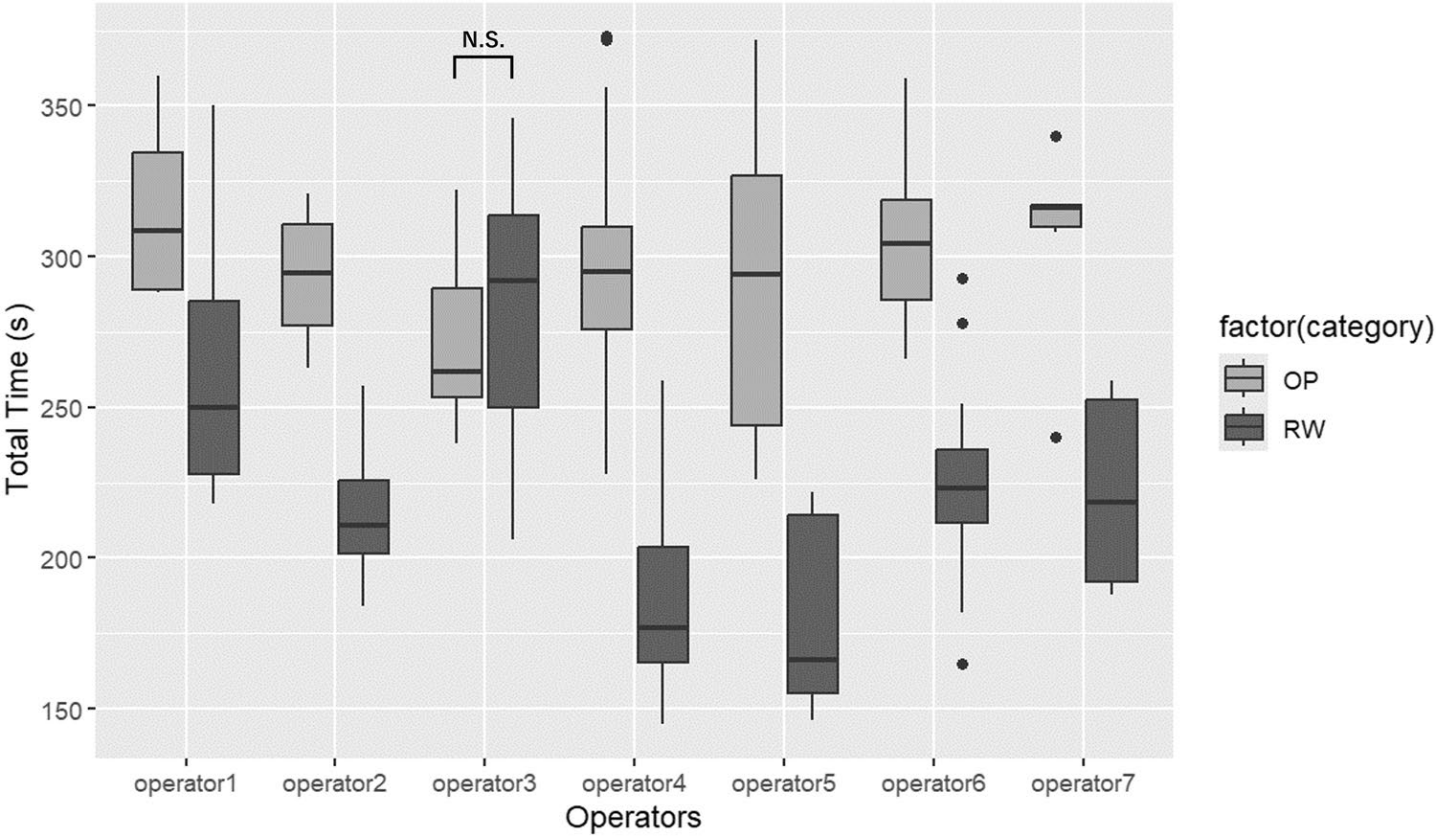
Comparison in operators. As indicated in Table 3, no significant correlation was found between the total processing time and operator 3 in the RW environment. In addition, there was no significant difference in the total processing time between the OP and RW environments for operator 3

No significant correlation was found in the OP environment regarding the relationship between operators and processing time. However, in the RW environment, all operators except for one achieved time reductions. The operator who did not achieve time reductions had longer operation times than the others, regardless of whether the OP or RW environment was used, and there was no significant difference between OP and RW for that operator (Table 4 and Fig. 5).

**Table 4:** Multiple regression analysis of operator versus processing time in OP and RW

Variables	*β*	SE	*t*	*p*
Results in OP environment				
Independent variable 1 (Operator2)	– – 21.500	19.511	– 1.102	0.27334
Independent variable 2 (Operator3)	– 40.483	13.089	– 3.093	0.00262
Independent variable 3 (Operator4)	– 22.368	11.347	– 1.971	0.05168
Independent variable 4 (Operator5)	– 20.528	14.902	– 1.378	0.17166
Independent variable 5 (Operator6)	– 7.500	12.340	– 0.608	0.54482
Independent variable 6 (Operator7)	– 8.583	16.897	– 0.508	0.61268
Intercept (Operator 1)	314.750	9.756	32.263	< 2e-16
Overall model: *R*^2^ = 0.0668, F(6, 93) = 2.181, *p* = 0.05164				
Results in RW environment				
Independent variable 1 (Operator2)	– 46.50	19.94	– 2.332	0.02186
Independent variable 2 (Operator3)	22.42	13.38	1.676	0.09712
Independent variable 3 (Operator4)	– 73.52	11.60	– 6.339	8.20e-09
Independent variable 4 (Operator5)	– 80.14	15.23	– 5.262	9.09e-07
Independent variable 5 (Operator6)	– 38.80	12.61	– 3.077	0.00275
Independent variable 6 (Operator7)	– 40.42	17.27	– 2.340	0.02140
Intercept (Operator 1)	262.25	9.97	26.304	< 2e-16
Overall model: *R*^2^ = 0.51, F(6, 93) = 18.23, *p* < 0.01

## Discussion

This study evaluated whether a dedicated image processing workstation installed at a remote location could be operated without performance degradation. The same examiner processed the same data in both the OP and RW environments, and the processing times for each task were measured. The overall impact on workflow was assessed over time. A comprehensive evaluation by all examiners showed that the RW environment reduced processing time by about 1 min. Initially, we anticipated that performance drawbacks would arise in tasks, such as re-slicing and rendering, which involve complex procedures, in the RW environment. However, despite data transfers and operations over a wide-area network (WAN), rendering performance was found to be equivalent to that of the OP environment. Furthermore, during the re-slicing of large image data sets, an average time reduction of 70 s was completed with the dedicated image processing device in the RW environment. This is likely because re-slicing requires processing the entire data set, which is a heavy task, demonstrating the advantage of the dedicated device. In actual workflow, the benefits are not limited to simple reductions in waiting time but also include a significant decrease in the time the device is occupied for processing. While local image processing devices also manage image transfer, image transfers may stop during intensive image processing, causing workflow interruptions. Using RW to distribute workloads could bring about additional workflow improvements that this study could not measure. In addition, while a dedicated image processing device is expensive and challenging to prepare specifically for the reconstruction of dental CBCT images, cost-effectiveness could be achieved using medical imaging devices during available time slots.

As a medical service utilizing networks, there are various applications such as inter-facility information sharing, cloud services for personal health records, or web services to assist in diagnosing specific diseases [4–7]. Systems for remote monitoring or image recognition using artificial intelligence have also been developed [8]. Cloud computing has become common for sharing devices with high processing power, such as computer assisted design or large-scale language models requiring complex data processing [9–11]. In medical imaging, high-performance processing devices are needed to handle large volumes of images with various modifications or analyses [12]; however, cloud-based services are still limited in the medical field [13], and while there is research on web services for 3D image processing in medicine [14, 15], they are not yet widely adopted. Services using the Remote Desktop Protocol (RDP) provide a solution to reduce network load when using devices installed remotely [16, 17], and it has been reported that performance can be adequately maintained in a local area network environment. In this study, we used a commercial network with a 2Gbps bandwidth, sharing it with all traffic in the medical information systems of two hospital facilities. This traffic includes electronic medical records and image viewing in diagnostics and the registration of image information from CT, MRI, endoscopy, and ultrasound examinations. Since remote operation uses a remote display protocol, the proportion of network bandwidth used is kept low. It could be used without delay, even in a shared environment with other systems, which is essential. Moreover, from the viewpoint of image processing devices, the local terminal is a thin client system. As long as it can run a remote display protocol, it can be used without being restricted by installation location or model, allowing work locations to be changed without physical or spatial constraints.

Within the scope of our search, we were unable to find any papers discussing the impact of environmental factors, such as internet speed and PC performance on medical imaging, comparisons across different applications, or the effects of physical distance on communication quality.

Theoretically, differences in PC performance among workstations affect processing time. In this study, the image processing environment used in RW typically exhibits higher performance than OP setups, with applications specifically designed for image processing, leading to an expected reduction in processing time. In addition, while the image sizes include data with varying FOV, the same data are processed and compared in both groups, effectively eliminating the influence of differences in data size.

Although there are studies on web-based architectures, we could not find any research evaluating the impact of inter-site distance in a configuration that directly utilizes a WAN as in this study. However, using RDP and a bandwidth-guaranteed line, we believe that sufficient communication quality was ensured, enabling real-time processing.

In summary, even when the image processing equipment is located at a remote site, it can be operated without compromising usability. The fact that workflow improvements are observed depending on the performance of the image processing equipment demonstrates that high-performance resources can be utilized via a WAN environment with performance equivalent to that of a LAN connection.

Limitations include the variation in processing times among examiners, the inability to fully evaluate the impact of other systems on network bandwidth usage, the use of a dedicated remote display protocol, and the fact that the image processing device used for remote access does not support multi-client simultaneous access. Regarding the variation in examiners' processing times, one specific examiner showed no significant difference in processing times between the OP and RW environments. This examiner generally had longer operation times than others, and the variation in processing times was considerable in the RW environment, suggesting a possible lack of proficiency in image processing operations.

While it is difficult to eliminate the impact of other systems' network traffic, the network utilization bandwidth is constantly monitored centrally, and based on the average values of network traffic every 2 h, the peak utilization during the day is about 20%, with an average of around 6%, suggesting that the impact of network bandwidth limitations is limited. In addition, to address network failures, we use a dualized 1Gbps line, which is expected to provide an environment with no logical restrictions due to bandwidth, even if one of the lines is completely down.

The dedicated remote display protocol could be replaced by the Windows OS standard RDP (winRDP), as suggested [16]. This would enable operation without restrictions on location or terminal type, although verification of winRDP-based performance in practical settings has not yet been conducted. The image processing device used for remote access does not support multi-client simultaneous access, which needs to be addressed in the future. Still, this is managed by checking machine availability through operational management.

In conclusion, dental CBCT image processing with a remote display protocol can be performed at a remote location with processing times equal to or less than those using on-premises devices, suggesting potential benefits for workflow improvement.
